# Patterns of Presentation and Histopathology of Retroperitoneal Sarcoma in a South Asian Tertiary Center

**DOI:** 10.7759/cureus.110556

**Published:** 2026-06-09

**Authors:** Hasnat Z Zim, Mitu Debnath, S K Mozammel Haque, Mohammad Ziaur Rahman, Hasan Shahrear Ahmed

**Affiliations:** 1 Department of General Surgery, Bangladesh Medical University, Dhaka, BGD; 2 Department of Surgery, Sher-E-Bangla Medical College Hospital, Barisal, BGD; 3 Department of Surgery, Bangladesh Medical University, Dhaka, BGD; 4 Department of Surgical Oncology, Bangladesh Medical University, Dhaka, BGD

**Keywords:** fnac, histopathology, liposarcoma, retroperitoneal sarcoma, surgery, surgical oncology

## Abstract

Background: Retroperitoneal sarcoma (RPS) is a rare and heterogeneous malignancy that often presents late due to its deep anatomical location and nonspecific symptoms. Data from South Asian settings remain limited.

Objective: This study aimed to describe the patterns of clinical presentation, diagnostic evaluation, and histopathological characteristics of surgically managed retroperitoneal sarcoma in a tertiary center in Bangladesh.

Methods: This descriptive observational case series was conducted in the Department of General Surgery (Surgical Oncology Unit) at the Bangladesh Medical University, Dhaka, from July 2021 to June 2022. Twenty-eight adult patients with histopathologically confirmed retroperitoneal sarcoma who underwent surgical management were included. Demographic data, presenting symptoms, imaging findings, histologic subtypes, tumor grade, margin status, and perioperative treatment were analyzed using descriptive statistics.

Results: The mean age was 45.4 ± 16.4 years, with a slight male predominance. Abdominal lump (78.6%) and pain (46.4%) were the most common presenting features. Contrast-enhanced CT was used in all cases, with a median tumor size of 12.75 cm on imaging. Liposarcoma subtypes predominated (50.0%), and most tumors were Federation of Cancer Centers Sarcoma Group grade 2 or 3. Complete resection with negative margins was achieved in 39.3%, while multivisceral resection was required in 25.0%. Adjuvant therapy was administered in 75.0% of patients, most commonly chemotherapy alone.

Conclusion: RPS in this cohort presented predominantly as large, locally advanced tumors with heterogeneous histopathology. Surgical resection remained the primary treatment modality, often requiring complex operative strategies. These findings highlight the need for early diagnosis and specialized multidisciplinary management in resource-limited settings.

## Introduction

Retroperitoneal sarcoma (RPS) is a rare and biologically heterogeneous subset of soft tissue sarcomas arising from mesenchymal tissues within the retroperitoneum, accounting for approximately 10%-15% of all soft tissue sarcomas and less than 1% of adult malignancies [1-3]. Although uncommon, RPS is clinically important because of its deep anatomical location, nonspecific presentation, histologic diversity, and complex management requirements [1]. The retroperitoneal compartment provides a large, relatively symptom-silent space that allows tumors to grow substantially before detection, often exceeding 10 cm in size by the time of diagnosis [4,5]. As a result, patients frequently present with vague abdominal symptoms, palpable mass, discomfort, or incidental imaging findings rather than early disease-specific features [4,6].

The clinical presentation of RPS is largely determined by tumor size, location, and involvement of adjacent organs. Abdominal lump, pain, gastrointestinal symptoms, urinary symptoms, and constitutional complaints may occur as a result of mass effect, while a subset of patients may remain asymptomatic until tumors are detected incidentally [2,4,5]. In routine clinical practice, abdominal ultrasonography is often the initial investigation performed for abdominal pain or a palpable abdominal mass, especially in resource-limited settings. However, contrast-enhanced computed tomography (CT) remains the cornerstone imaging modality for further evaluation of suspected RPS, as it provides essential information regarding tumor size, anatomical extent, vascular involvement, relation to adjacent organs, and surgical resectability [7,8]. Magnetic resonance imaging (MRI) may provide additional soft tissue detail in selected cases, particularly for pelvic or complex lesions, but its use may be limited by availability and cost [7]. Despite these imaging advances, radiologic assessment alone may not reliably distinguish benign from malignant retroperitoneal masses or define histologic subtype, making histopathologic confirmation essential [4,7].

Histopathologic evaluation is central to the diagnosis and classification of RPS. The major subtypes include well-differentiated and dedifferentiated liposarcoma, leiomyosarcoma, undifferentiated pleomorphic sarcoma, solitary fibrous tumor, and malignant peripheral nerve sheath tumor [9,10]. These subtypes differ in biological behavior, recurrence pattern, metastatic potential, and prognosis [11]. Histologic grading systems, including the French Federation of Cancer Centers Sarcoma Group (FNCLCC) criteria, further support prognostic assessment by incorporating tumor differentiation, mitotic activity, and necrosis [10]. Therefore, accurate histologic classification and grading are important for treatment planning, prognostication, and multidisciplinary decision-making [9,10].

International recommendations emphasize multidisciplinary evaluation of RPS, integrating surgery, radiology, pathology, and oncology [4,11]. Complete surgical resection remains the only potentially curative treatment, but achieving clear margins can be difficult because of the close relationship of these tumors to major vessels, kidneys, bowel, and other retroperitoneal structures [1,4]. In high-volume sarcoma centers, standardized imaging, tissue diagnosis, specialist pathology, and tumor board-based decision-making guide management [6,11]. However, these pathways may not be fully reproducible in low- and middle-income countries, where delayed presentation, restricted access to advanced imaging and immunohistochemistry, and limited sarcoma-specific expertise may affect diagnostic evaluation and treatment planning [5].

Data on RPS from South Asian tertiary centers remain limited, particularly regarding local patterns of clinical presentation, diagnostic evaluation, imaging findings, histopathological profile, and perioperative management. Locally generated observational data are therefore important for understanding how RPS presents and is managed in resource-limited referral settings, and for identifying practical gaps in diagnostic and treatment pathways. Against this background, the present study aimed to describe the baseline characteristics, clinical presentation, diagnostic evaluation, histopathological features, surgical approaches, and perioperative treatment patterns of patients with retroperitoneal sarcoma managed at a tertiary referral center in Bangladesh.

## Materials and methods

This descriptive observational case series study was conducted at the Department of General Surgery (Surgical Oncology Unit), Bangladesh Medical University, Dhaka, Bangladesh, from July 2021 to June 2022. All consecutive patients with retroperitoneal sarcoma who were managed surgically during the study period were considered for inclusion. Adult patients of either sex with radiologically suspected retroperitoneal tumors who underwent surgical exploration and subsequently had histopathological confirmation of retroperitoneal sarcoma were included in the study. Only surgically managed patients with histopathologically confirmed retroperitoneal sarcoma were eligible for inclusion. Patients with recurrent disease previously treated elsewhere without complete clinical records, patients without confirmatory histopathology, and patients with unresectable disease who did not undergo surgery were excluded. A total of 28 patients constituted the final study cohort.

Demographic characteristics, presenting symptoms, clinical history, and relevant investigation findings were collected from patient interviews and hospital records. Preoperative diagnostic evaluation included contrast-enhanced CT in all patients. Magnetic resonance imaging (MRI), fine-needle aspiration cytology (FNAC), and core needle biopsy were performed selectively according to clinical indication, feasibility, and resource availability. Immunohistochemistry was performed selectively in diagnostically challenging cases according to pathologist discretion and resource availability. Diagnostic delay was defined as the interval between presentation and establishment of diagnosis, while preoperative interval was defined as the interval between hospital admission and definitive surgery.

Operative findings, extent of resection, adjacent organ involvement, and perioperative treatment details were recorded. Resected specimens were examined in the Department of Pathology, Bangabandhu Sheikh Mujib Medical University, Dhaka. Histologic subtyping was performed according to standard morphologic criteria, and tumor grading was assigned using the French FNCLCC grading system where applicable. Resection margins were classified as R0, R1, or R2 according to histopathology reports, while cases with incomplete margin documentation were recorded as not assessed. Tumor size was documented from both imaging and histopathological measurements, and size categories were derived according to the American Joint Committee on Cancer (AJCC) soft tissue sarcoma staging criteria. Concordance between preoperative investigations and final histopathological diagnosis was assessed by comparing the diagnostic impressions of CT imaging, FNAC, and core needle biopsy with the definitive postoperative histopathological diagnosis.

Data were checked for completeness and analyzed using Statistical Package for the Social Sciences (SPSS) version 22.0 (IBM Corp., Armonk, NY). Continuous variables were summarized using medians and ranges, while categorical variables were presented as frequencies and percentages. Ethical approval was obtained from the Institutional Review Board of Bangabandhu Sheikh Mujib Medical University, and written informed consent was obtained from all participants before inclusion in the study.

## Results

Table 1 summarizes the baseline demographic and clinical characteristics of the 28 patients with retroperitoneal sarcoma included in the study. The mean age of the cohort was 45.4 ± 16.4 years, with a median age of 45 years, ranging from 18 to 71 years. Male patients constituted a slight majority, accounting for 57.1% of the study population, while female patients represented 42.9%. Most patients belonged to the middle socioeconomic group (64.3%), followed by those from lower socioeconomic backgrounds (28.6%), with a small proportion classified as high socioeconomic status (7.1%). More than half of the patients were nonsmokers (53.6%), whereas 46.4% reported a history of smoking. A family history of relevant malignancy was uncommon, being documented in only one patient (3.6%), while the remaining 96.4% reported no such history.

**Table 1:** Baseline characteristics of patients with retroperitoneal sarcoma (n = 28)

Characteristics	Value
Age	
Mean ± SD (years)	45.4 ± 16.4
Median (range), years	45.0 (18-71)
Sex, n (%)	
Male	16 (57.1)
Female	12 (42.9)
Socioeconomic condition, n (%)	
Middle	18 (64.3)
Low	8 (28.6)
High	2 (7.1)
Smoking status, n (%)	
No	15 (53.6)
Yes	13 (46.4)
Family history of relevant malignancy, n (%)	
Absent	27 (96.4)
Present	1 (3.6)

Table 2 outlines the patterns of clinical presentation and pathway intervals among the study population. The majority of patients presented with an abdominal lump or palpable mass, reported in 78.6% of cases, making it the most frequent presenting feature. Abdominal pain was noted in nearly half of the patients (46.4%). A smaller proportion of patients were asymptomatic at presentation (14.3%). Constitutional and systemic symptoms were less common, with weight loss reported by 25.0% of patients, anorexia by 17.9%, and fever by 3.6%. Gastrointestinal symptoms were present in 25.0% of cases and urinary symptoms in 10.7%. Other nonspecific symptoms were reported in 7.1% of patients. The median admission-to-surgery interval was 17.5 days, with a range of 14.0-33.0 days, reflecting the time between hospital admission and definitive surgical management.

**Table 2:** Patterns of presentation and pathway intervals (n = 28)

Symptom or feature	Value
Asymptomatic at presentation, n (%)	4 (14.3)
Abdominal lump/mass, n (%)	22 (78.6)
Abdominal pain, n (%)	13 (46.4)
Anorexia, n (%)	5 (17.9)
Weight loss, n (%)	7 (25.0)
Fever, n (%)	1 (3.6)
Gastrointestinal symptoms, n (%)	7 (25.0)
Urinary symptoms, n (%)	3 (10.7)
Other symptoms, n (%)	2 (7.1)
Admission-to-surgery interval, days, median (range)	17.5 (14.0-33.0)

**Figure 1:**
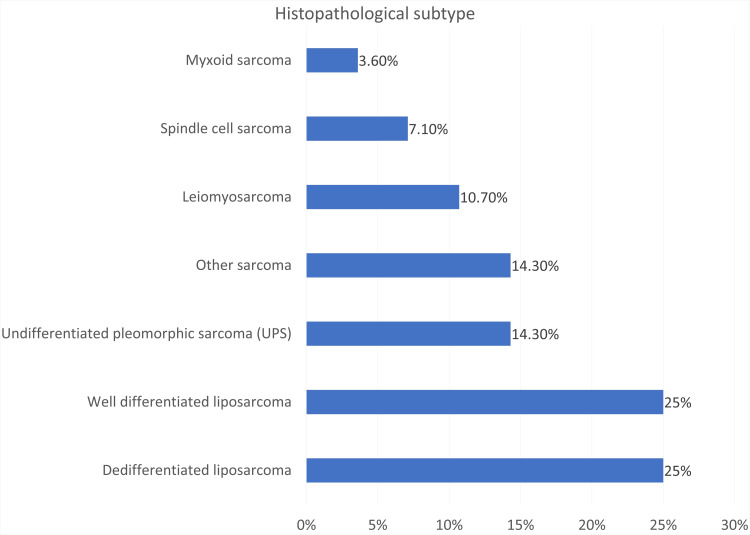
Distribution of histopathologic subtypes (n = 28)

Figure 1 demonstrates the distribution of histopathologic subtypes among the 28 retroperitoneal sarcoma cases. Dedifferentiated liposarcoma and well-differentiated liposarcoma were the most frequent subtypes, each accounting for seven patients (25.0%). Undifferentiated pleomorphic sarcoma (UPS) and the category labeled as other sarcoma were each observed in four patients (14.3%). Leiomyosarcoma was identified in three patients (10.7%), spindle cell sarcoma in two patients (7.1%), and myxoid sarcoma in one patient (3.6%), indicating a predominance of liposarcoma subtypes with a smaller representation of other histologic variants.

Table 3 presents the preoperative diagnostic workup and imaging characteristics at initial presentation. Contrast-enhanced CT was performed in all patients (100.0%), serving as the primary imaging modality, while magnetic resonance imaging was used selectively in two patients (7.1%). Preoperative tissue diagnosis was obtained in a limited proportion of cases, with fine-needle aspiration cytology performed in 25.0% and core needle biopsy in 7.1% of patients. Immunohistochemistry was utilized in two cases (7.1%). On imaging, the most common tumor location was the left lower quadrant, observed in 32.1% of patients, followed by pelvic tumors in 21.4%. Right upper quadrant tumors accounted for 17.9% of cases, while left upper and right lower quadrant locations were each noted in 14.3%. The median tumor size on imaging was 12.75 cm, with a wide range from 4.90 to 48.00 cm. Imaging evidence of contiguous organ involvement was present in 35.7% of patients, whereas distant metastasis at presentation was uncommon, identified in only one case (3.6%).

**Table 3:** Preoperative diagnostic workup and imaging characteristics at presentation (n = 28) FNAC: fine-needle aspiration cytology; CT: computed tomography; MRI: magnetic resonance imaging

Characteristic	Value
FNAC performed, n (%)	7 (25.0)
Core needle biopsy performed, n (%)	2 (7.1)
CT scan performed, n (%)	28 (100.0)
MRI performed, n (%)	2 (7.1)
Immunohistochemistry performed, n (%)	2 (7.1)
Tumor localization on imaging, n (%)	
Left lower quadrant	9 (32.1)
Pelvis	6 (21.4)
Right upper quadrant	5 (17.9)
Left upper quadrant	4 (14.3)
Right lower quadrant	4 (14.3)
Tumor size on imaging, cm, median (range)	12.75 (4.90-48.00)
Organ involvement on imaging, n (%)	10 (35.7)
Distant metastasis on imaging, n (%)	1 (3.6)

Table 4 summarizes the histopathological characteristics, tumor grade, resection margin status, and size categories of the study cohort. The median histopathologic tumor size was 14.5 cm, with values ranging from 5 to 49 cm. Dedifferentiated liposarcoma and well-differentiated liposarcoma were the most frequently identified histologic subtypes, each comprising 25.0% of cases, followed by undifferentiated pleomorphic sarcoma and other sarcoma subtypes, each accounting for 14.3%. Leiomyosarcoma was observed in 10.7% of patients, while spindle cell sarcoma and myxoid sarcoma were less common. Based on FNCLCC grading, grade 2 tumors were most prevalent, representing 42.9% of cases, followed by grade 3 tumors in 35.7% and grade 1 tumors in 21.4%. Complete resection with negative margins (R0) was achieved in 39.3% of patients, whereas positive microscopic (R1) and grossly positive (R2) margins were documented in 17.9% and 10.7% of cases, respectively; margin status was not assessed or unavailable in 32.1% of patients. According to AJCC tumor size categories, nearly half of the tumors were classified as T4 (>15 cm), accounting for 46.4% of cases, while T3, T2, and T1 tumors constituted 25.0%, 21.4%, and 7.1% of the cohort, respectively.

**Table 4:** Histopathological characteristics, grade, margin status, and size category (n = 28) FNCLCC: French Federation of Cancer Centers Sarcoma Group; AJCC: American Joint Committee on Cancer

Characteristic	Value
Histopathologic tumor size, cm, median (range)	14.50 (5-49)
Histologic subtype, n (%)	
Dedifferentiated liposarcoma	7 (25.0)
Well differentiated liposarcoma	7 (25.0)
Undifferentiated pleomorphic sarcoma	4 (14.3)
Other sarcoma	4 (14.3)
Leiomyosarcoma	3 (10.7)
Spindle cell sarcoma	2 (7.1)
Myxoid sarcoma	1 (3.6)
FNCLCC grade, n (%)	
Grade 1	6 (21.4)
Grade 2	12 (42.9)
Grade 3	10 (35.7)
Resection margin status, n (%)	
R0	11 (39.3)
Missing/not assessed	9 (32.1)
R1	5 (17.9)
R2	3 (10.7)
Tumor size category (AJCC T), n (%)	
T4 (>15 cm)	13 (46.4)
T3 (>10-15 cm)	7 (25.0)
T2 (>5-10 cm)	6 (21.4)
T1 (≤5 cm)	2 (7.1)

Table 5 outlines the surgical approaches employed and the patterns of perioperative therapy among the study population. Simple complete tumor resection without removal of adjacent organs was the most common surgical procedure, performed in 64.3% of patients. Contiguous organ resection as part of a multivisceral approach was required in 25.0% of cases, while gross residual disease following surgery was documented in 10.7% of patients. Among those undergoing multivisceral resection, involvement of the colon and diaphragm was the most frequently resected contiguous organs, each in 7.1% of patients, followed by resection of the psoas fascia, psoas muscle, and other structures in smaller proportions. With regard to perioperative therapy, neoadjuvant treatment was administered in 10.7% of patients, whereas adjuvant therapy was given to the majority of the cohort (75.0%). Chemotherapy alone was the most common adjuvant modality, received by 64.3% of patients, while combined chemotherapy and radiotherapy was administered in 10.7%, and no adjuvant therapy was given in 25.0% of cases.

**Table 5:** Surgical approach and perioperative therapy patterns (n = 28)

Characteristic	Value
Type of surgery, n (%)	
Simple complete resection	18 (64.3)
Contiguously involved organ resection	7 (25.0)
Gross residual disease	3 (10.7)
Contiguous organ resection details, n (%)	
Any contiguous organ resection (multivisceral)	7 (25.0)
Colon	2 (7.1)
Diaphragm	2 (7.1)
Psoas fascia	1 (3.6)
Psoas muscle	1 (3.6)
Others	1 (3.6)
Perioperative therapy, n (%)	
Neoadjuvant therapy received	3 (10.7)
Adjuvant therapy received	21 (75.0)
Type of adjuvant therapy, n (%)	
Chemotherapy	18 (64.3)
Chemotherapy + radiotherapy	3 (10.7)
Not given	7 (25.0)

## Discussion

Retroperitoneal sarcoma (RPS) is an uncommon and biologically heterogeneous malignancy that presents unique diagnostic and therapeutic challenges. Owing to the large potential space of the retroperitoneum and the absence of early disease-specific symptoms, tumors frequently attain considerable size before diagnosis, often resulting in locally advanced disease at presentation [1,4]. The present study provides a real-world overview of the preoperative diagnostic workup and clinicopathological characteristics of surgically managed RPS in a tertiary referral center in Bangladesh, highlighting both similarities and differences compared with reports from specialized sarcoma centers.

The demographic profile of the study population was broadly consistent with international experience. Most patients were middle-aged adults, and the sex distribution was relatively balanced, findings comparable to those reported in large institutional and multidisciplinary series [2,4]. The predominance of patients from middle and lower socioeconomic groups may reflect disparities in healthcare access, referral pathways, and diagnostic availability, factors that have been recognized as contributors to delayed cancer diagnosis in resource-constrained settings [2,4]. Family history of malignancy was uncommon, consistent with the predominantly sporadic nature of RPS reported worldwide [1].

A notable finding of the present study was the prolonged diagnostic pathway. The median diagnostic delay was 45 days, while the median interval between admission and surgery was 17.5 days. Although direct comparison is difficult because such metrics are infrequently reported in RPS studies, these findings support concerns regarding delays in diagnosis and treatment that may occur in settings where access to specialized imaging, image-guided biopsy, advanced pathology services, and multidisciplinary sarcoma care is limited. Because RPS often remains clinically silent until substantial growth has occurred, even modest delays may contribute to increased tumor burden and greater surgical complexity at presentation [4,5].

Abdominal lump and abdominal pain were the predominant presenting symptoms, reflecting the classical “mass-effect” presentation of retroperitoneal tumors. Similar observations have been reported in imaging and surgical series, where symptoms generally arise from displacement or compression of adjacent structures rather than direct tumor invasion [2,8]. The relatively low proportion of constitutional symptoms and the presence of a small asymptomatic subgroup further support previous observations that RPS frequently remains undetected until identified clinically or incidentally through imaging investigations [4,8].

The present study also provides insight into preoperative diagnostic practices in a resource-limited environment. Contrast-enhanced CT was performed in all patients and represented the principal diagnostic modality, consistent with international recommendations identifying CT as the cornerstone investigation for retroperitoneal masses [8,11]. In contrast, MRI was used infrequently, reflecting both selective clinical indications and practical constraints related to cost and availability. Preoperative tissue diagnosis was similarly limited, with only a minority of patients undergoing FNAC or core needle biopsy. Contemporary guidelines generally advocate image-guided core needle biopsy before treatment because it facilitates histologic diagnosis, multidisciplinary planning, and treatment selection [4,11]. However, the low uptake observed in this study likely reflects real-world limitations commonly encountered in low-resource settings, including restricted access to interventional radiology, logistical barriers, and financial constraints. These findings emphasize the gap that may exist between guideline-recommended practice and actual implementation in developing healthcare systems.

Assessment of concordance between preoperative investigations and final histopathology demonstrated variable diagnostic performance across modalities. CT correctly identified retroperitoneal sarcoma in the majority of cases, supporting its utility as an initial diagnostic tool. However, imaging alone cannot reliably establish histologic subtype, grade, or definitive diagnosis, reinforcing the continued importance of tissue confirmation. FNAC demonstrated lower concordance than CT and core biopsy, which is consistent with existing evidence indicating that cytology alone may be insufficient for accurate sarcoma classification. Histopathological examination therefore remains the diagnostic gold standard for RPS, while immunohistochemistry serves as an important adjunct for subtype differentiation and diagnostic confirmation in selected cases [9,10]. The low utilization of immunohistochemistry in this series likely reflects resource limitations rather than reduced clinical importance.

The histopathological findings observed in this study were largely comparable to those reported in major international series. Liposarcoma, including both well-differentiated and dedifferentiated variants, was the most common subtype, followed by undifferentiated pleomorphic sarcoma and leiomyosarcoma [6,11,12]. This distribution mirrors reports from Europe, North America, and Asia, where liposarcoma consistently accounts for the largest proportion of retroperitoneal sarcomas [1,3]. Furthermore, most tumors were classified as FNCLCC grade 2 or grade 3, reflecting the predominance of intermediate- and high-grade lesions that characterize many surgically treated RPS cohorts [1]. These findings underscore the marked histological heterogeneity of RPS and the importance of accurate pathological assessment.

Tumors in this cohort were generally large at diagnosis, with median radiologic and pathological dimensions exceeding 12 and 14 cm, respectively. Nearly half of the patients had AJCC T4 disease, supporting previous observations that RPS frequently presents as bulky, locally advanced tumors [1,4]. Organ involvement was common, and multivisceral resection was required in a substantial proportion of cases. These findings are consistent with reports from specialized sarcoma centers, where complete resection often necessitates removal of adjacent organs to achieve optimal local control [13,14]. Although R0 resection remains the preferred surgical goal, the anatomical complexity of the retroperitoneum often makes negative margins difficult to achieve.

Adjuvant treatment patterns in this study demonstrated considerable variability. Most patients received postoperative chemotherapy, while a smaller proportion underwent combined chemoradiotherapy or observation alone. Detailed information regarding individual chemotherapy regimens was unavailable in the clinical records and therefore could not be evaluated. In contemporary practice, commonly utilized systemic agents for advanced or high-risk soft tissue sarcomas include doxorubicin, ifosfamide, gemcitabine, docetaxel, trabectedin, and eribulin, depending on histologic subtype and treatment intent. Nevertheless, the role of adjuvant chemotherapy in resected retroperitoneal sarcoma remains controversial. Recent systematic reviews and meta-analyses have demonstrated limited evidence for improvements in overall survival or recurrence-free survival following routine adjuvant chemotherapy [15,16]. Consequently, treatment decisions are often individualized according to tumor subtype, grade, resection status, and institutional practice patterns. The variability observed in the present study is therefore consistent with the broader uncertainty that continues to characterize systemic treatment of RPS.

Several limitations should be considered when interpreting these findings. The study was conducted at a single tertiary referral center and included a relatively small sample size, limiting external generalizability. Only surgically managed patients were included; therefore, patients with unresectable, metastatic, or nonoperatively managed disease were not represented. This may have introduced selection bias toward patients considered suitable for surgical treatment. In addition, detailed immunohistochemical profiles, chemotherapy regimens, treatment-response data, and long-term oncologic outcomes were unavailable, limiting more comprehensive evaluation of prognostic factors and treatment effectiveness. Despite these limitations, the study provides valuable information regarding the real-world diagnostic workup and clinicopathological characteristics of retroperitoneal sarcoma in a resource-limited tertiary care setting and contributes region-specific evidence to a field dominated by reports from high-volume centers in high-income countries.

## Conclusions

Retroperitoneal sarcoma in this South Asian tertiary center predominantly presented in middle adulthood with large, locally advanced tumors, most commonly of liposarcoma histology. Clinical presentation was largely driven by mass effect, with abdominal lump and pain as the principal symptoms, while distant metastasis at diagnosis was uncommon. Imaging, particularly contrast-enhanced CT, played a central role in diagnosis and surgical planning, and complete resection remained the cornerstone of management, often requiring multivisceral surgery. The predominance of intermediate- to high-grade tumors and the frequent use of adjuvant chemotherapy reflect both disease biology and real-world practice patterns. These findings underscore the importance of early recognition, meticulous surgical planning, and context-appropriate multidisciplinary care in improving outcomes for patients with retroperitoneal sarcoma.
